# Expansion of mass-flowering crops leads to transient pollinator dilution and reduced wild plant pollination

**DOI:** 10.1098/rspb.2011.0268

**Published:** 2011-04-06

**Authors:** Andrea Holzschuh, Carsten F. Dormann, Teja Tscharntke, Ingolf Steffan-Dewenter

**Affiliations:** 1 Agroecology, Department of Crop Sciences, Georg-August University, Grisebachstr. 6, 37077 Göttingen, Germany; 2 Department of Animal Ecology and Tropical Biology, Biocenter, University of Würzburg, Am Hubland, 97074 Würzburg, Germany; 3 Department of Computational Landscape Ecology, UFZ Helmholtz Centre for Environmental Research, Permoserstraße 15, 04318 Leipzig, Germany

**Keywords:** canola, competition, facilitation, oilseed rape, pollination, spill-over

## Abstract

Agricultural land use results in direct biodiversity decline through loss of natural habitat, but may also cause indirect cross-habitat effects on conservation areas. We conducted three landscape-scale field studies on 67 sites to test the hypothesis that mass flowering of oilseed rape (*Brassica napus*) results in a transient dilution of bees in crop fields, and in increased competition between crop plants and grassland plants for pollinators. Abundances of bumble-bees, which are the main pollinators of the grassland plant *Primula veris*, but also pollinate oilseed rape (OSR), decreased with increasing amount of OSR. This landscape-scale dilution affected bumble-bee abundances strongly in OSR fields and marginally in grasslands, where bumble-bee abundances were generally low at the time of *Primula* flowering. Seed set of *Primula veris*, which flowers during OSR bloom, was reduced by 20 per cent when the amount of OSR within 1 km radius increased from 0 to 15 per cent. Hence, the current expansion of bee-attractive biofuel crops results in transient dilution of crop pollinators, which means an increased competition for pollinators between crops and wild plants. In conclusion, mass-flowering crops potentially threaten fitness of concurrently flowering wild plants in conservation areas, despite the fact that, in the long run, mass-flowering crops can enhance abundances of generalist pollinators and their pollination service.

## 1. Introduction

Negative consequences of land-use intensification and habitat loss for biodiversity and associated ecosystem services have often been reported [1–3], but the exact mechanisms are still poorly understood [4]. Although biodiversity loss is mostly assumed to be a direct result of decreasing habitat area and of impeded organism exchanges between habitat fragments [5,6], indirect effects mediated by changed species interactions might be just as important [7,8].

Indirect effects of land-use intensification via species interactions can be expected to be ubiquitous where managed and natural habitats adjoin, or where species using multiple habitats connect managed and natural habitats on a larger scale [9,10]. Changes in species interactions might occur everywhere in mosaic landscapes where (i) at least one of two interacting partners uses both managed and natural habitats, and where (ii) land-use intensification (i.e. the difference between managed and natural habitats) affects one interaction partner more strongly than the other (positively or negatively).

There are several examples showing that mobile organisms occurring in managed habitats benefit from neighbouring natural habitats, which provide, for example, nesting sites or refuge after disturbances [11,12]. By contrast, evidence for organism spill-over from managed to natural habitats is extremely rare and consequences for species interactions are poorly known [13]. Spill-over from managed to natural habitats occurs if organisms benefit from the high productivity of managed habitats and then move to (semi-)natural habitats, which are normally less productive [14]. Predators and herbivores, which are subsidized by resources in managed habitats, can subsequently strongly affect prey species and plants in natural habitats [15]. Comparable but currently unknown changes of species interactions are possible for pollinators that visit highly productive mass-flowering crops for pollen and nectar instead of foraging and providing pollination in their semi-natural nesting habitats [7].

A preference of native bees for crop fields over semi-natural habitats might have serious negative effects for seed or fruit set of bee-pollinated wild plants in conservation areas. Competition between plant species for a limited number of pollinators can result in reduced flower visitation rates and reduced seed set in the less attractive species [16]. On the other hand, attractive plant species can also enhance visitation rates of neighbouring plants if the attractive plant attracts pollinators to a certain flower patch and pollinators subsequently visit all flowers within the patch independently of their attractiveness (‘good neighbours’ [16,17]). In that case, the positive effect of facilitation by the attractive magnet plant exceeds the negative effect of competition. The meta-analysis of Morales & Traveset [18] revealed that detrimental effects of invading plant species on pollination and reproduction of natives are predominant. While there are increasing numbers of field-scale studies focusing on interactions between simultaneously flowering plant species growing in a shared habitat [18–20], there are—as yet—no studies focusing on links among sites or habitat types via shared pollinators.

We conducted three large-scale field studies on 67 study sites to assess interactions between mass-flowering oilseed rape (OSR) and semi-natural grasslands, and their potential negative or positive effects on wild plants and bees. Pollen and nectar provided by OSR are highly attractive to bees. OSR is planted at a density of 350 000–700 000 plants per hectare, producing more than 100 times as many flowers per hectare [21]. The enormous flower density and the good accessibility of nectar and pollen facilitate a high number of flower visits per time unit. On forage trips, bumble-bees visit on average over 400 OSR flowers per visit and approximately 2000 flowers per hour [21].

**Figure 1.**
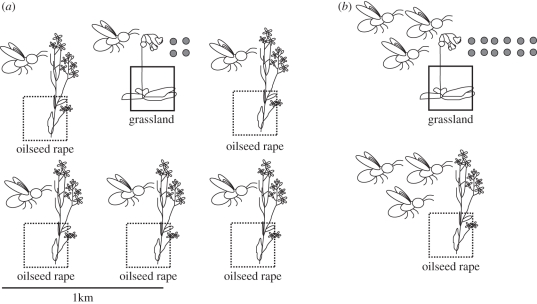
Landscape-scale dilution of bees in oilseed rape, and consequences for pollinator abundances and seed set. The number of grey dots indicates the number of seeds produced by a grassland plant. (*a*) High amount of oilseed rape results in high dilution of pollinators, low pollinator abundances per site and low reproduction of pollinator-dependent grassland plants. (*b*) Low amount of oilseed rape results in high pollinator abundances per site and high reproduction of pollinator-dependent grassland plants. Effects on oilseed rape production have not been studied here and hence its seed production is not indicated.

In study 1, we hypothesized that OSR enhances the diversity and abundances of bees in grasslands if the OSR fields directly adjoin the grassland. The large amount of additional flower resources provided by OSR fields can be expected to attract bees to nesting sites in the grassland (local scale effect). High amounts of OSR at the landscape scale were hypothesized to result in a transient dilution of bees during mass flowering, and to decrease bee abundances in grasslands (figure 1).

In study 2, we hypothesized that bee diversity and abundance in OSR are positively affected by adjacent grasslands, which provide nesting sites, and negatively by high amounts of OSR, which cause landscape-scale dilution effects (figure 1).

In study 3, we formulated two contrasting hypotheses. The facilitation hypothesis states that OSR enhances sexual reproduction of grassland plants, because their pollinators prefer to nest in grasslands adjacent to OSR, resulting in positive effects on the pollination of the grassland plants. The competition hypothesis states that OSR reduces the reproduction of grassland plants, because mass-flowering OSR is a superior competitor in attracting wild bees (figure 1).

## 2. Methods

### (a) Study sites

Three studies were carried out in 2007 near the town of Göttingen (51.5° N, 9.9° E), Lower Saxony, Germany. The study area is composed of intensively managed agricultural areas dominated by annual crop fields, and patchily distributed fragments of forests and semi-natural habitats such as calcareous grasslands. Calcareous grasslands belong to the most species-rich bee habitats in Europe [22] and are protected by law as conservation areas.

In an area of 25 × 30 km, we selected 67 study sites (33 calcareous grasslands and 34 *Brassica napus* OSR fields) belonging to four categories: (i) 16 grasslands were isolated by at least 230 m from OSR; (ii) 17 grasslands were at 1–15 m distance of OSR; (iii) 17 OSR fields were isolated by at least 570 m from calcareous grasslands; and (iv) 17 OSR fields were at 1–15 m distance of the study grasslands. Further details are given in the electronic supplementary material, appendix S1.

### (b) Landscape parameters

For each study site, the surrounding landscape was characterized in a landscape circle of 1 km radius. OSR fields, semi-natural habitats (calcareous grasslands, orchard meadows, old fallows, hedgerows) and other habitat types were mapped in the field and included into digital thematic maps. Geographic Information Systems (ESRI ARC/View v. 3.2) were used to calculate the proportion of each habitat type in the landscape sectors. The percentage of area covered with OSR (hereafter referred to as %OSR) spanned a gradient from 0 to 30.5 per cent and was not correlated with any other habitat type (Spearman rank correlations, all *p* > 0.1, *n* = 67). The percentage area covered with semi-natural habitat (%semi-natural habitat) spanned a gradient from 0.6 to 12.9 per cent, and was negatively correlated with the percentage of arable land and positively correlated with the Shannon index of habitat diversity calculated using the percentage of each habitat type (Spearman rank correlations, all *p* < 0.05, *n* = 67).

### (c) Study 1: bees in semi-natural grasslands

In grasslands, bees (Apiformes) were recorded during OSR flowering in April and May for 15 min in a 0.1 ha plot along a variable transect (following Westphal *et al*. [23]). Flower cover (percentage cover of flower corollas per area ground surface) and the number of plant species flowering during the survey in the 0.1 ha plot were recorded (number of plant species was missing for one of the grasslands adjacent to OSR). Bee abundances are expressed as bee densities per 100 m^2^ and 15 min. The diversity of bees equals the total number of solitary bee species. All bees that could not be identified in the field were collected for subsequent identification in the laboratory. Sites were sampled between 10.00 and 17.00 h under sunny weather conditions only (temperature greater than 16°C, cloud cover less than 10%, low wind speeds less than 2 Bft).

### (d) Study 2: bees in oilseed rape

Transect walks were conducted to assess bee diversity and abundance in OSR. Bees were recorded along 100 m transects with 1 m width in the field centre and at the field edge for 15 min per transect on two occasions during OSR flowering in April and May (2 × 15 min × 2 = 60 min per field). The diversity of bees equals the total number of bee species. The edge transect was located 1 m into the OSR field along the field edge; the centre transect started 10 m from the field edge and followed a lane into the direction of the field centre. Data from the four transect walks per OSR field were pooled for analysis. Bee abundances are expressed as bee densities per 400 m^2^ and 60 min.

### (e) Study 3: seed set of *Primula veris*

To assess the effect of OSR on the pollination and reproductive success of a grassland plant, we measured seed set of the cowslip *Primula veris* L. (Primulaceae). Flowering is usually during the period of OSR flowering, starting at the end of April and ending three to four weeks later. *Primula veris* is strictly self-incompatible and only cross-pollination between pin morphs (long pistil, short stamens) and thrum morphs (short pistil, long stamens) results in effective seed set [24]. Flowers are successfully pollinated by long-tongued bees (mainly bees of the genera *Bombus* and *Anthophora*) and bombyliid flies, which are able to exploit the nectar [25,26]. Because of the fragmentation of its habitat, *P. veris* is included in the Red Data Book as ‘critically endangered’ in Lower Saxony [27].

*Primula veris* occurred in 19 of the 33 study grasslands (7 adjacent to OSR, 12 isolated from OSR). At the beginning of flowering, we randomly marked 10–24 plants per grassland that all had one stalk. Some of the inflorescences were lost to mammal herbivory, resulting in 260 marked plants (155 plants adjacent to OSR, 105 plants isolated from OSR; mean per grassland ± s.d.: 13 ± 5.6, min: 2, max: 24) at the time of seed ripening. We recorded the number of plant species flowering in the 0.1 ha plot during the survey, and the flower cover of these species (percentage cover of flower corollas per area ground surface in the 0.1 ha plot). The number of *P. veris* individuals in the flower patch around the marked plants was recorded (patch edges were defined by a separation of greater than 3 m to the next conspecific individual). This parameter was correlated with patch size (Spearman rank correlation: *R* = 0.89, *p* < 0.001, *n* = 19) and with flower cover of *P. veris* in the 0.1 ha transect plots (*R* = 0.69, *p* = 0.003, *n* = 19). Morph ratios (pin : thrum morph) were not skewed in the study grasslands, and did not differ between isolated grasslands and grasslands adjacent to OSR (see the electronic supplementary material, appendix S2).

In July, the ripe fruits were collected and dried. The number of seeds per plant was counted and the seeds were weighed. Seed number per plant and seed weight per plant were divided by the total number of flowers minus the number of predated fruits (=number of intact fruits) to correct for differences in flower numbers and predation rate between plants (mean ± s.e. of non-predated fruits per plant: 5 ± 3). If seeds were predated by insect larvae, the fruit was excluded from the analyses, because usually all seeds were damaged and turned into crumbs. The predation rate was not related to the number of seeds per fruit (*R* = 0.11, *p* > 0.01, *n* = 19). The number and weight of seeds per fruit were taken as a measure of reproductive success and averaged over the plants of the study site.

### (f) Statistical analyses

Local and landscape effects on bees were assessed in ANCOVAs (type II sums of squares [28]). Response variables in grasslands were the diversity and abundances of solitary bees, and the abundance of bumble-bees. Presence–absence data for honeybees in grasslands were assessed in a logistic regression. Response variables in OSR were bee diversity, and abundances of solitary bees, bumble-bees and honeybees. Predictors for bees in grasslands were the presence of adjacent OSR (grasslands adjacent to OSR versus grasslands isolated from OSR), %OSR and %semi-natural habitats in 1 km radius, flower cover, diversity of flowering plant species, and interactions between presence of OSR at the local scale and the other factors. Predictors for bees in OSR were the presence of adjacent grassland (OSR adjacent to grassland versus OSR isolated from grassland), %OSR fields and %semi-natural habitats in 1 km radius, OSR field size, and interactions between presence of adjacent grassland and the other factors.

Effects on *P. veris* seed set were assessed in ANCOVAs with dependent variables the mean number of seeds per fruit and mean seed weight per fruit. Predictors were the presence of adjacent OSR (grasslands adjacent to OSR versus grasslands isolated from OSR), %OSR fields and %semi-natural habitats in 1 km radius, the number of *P. veris* individuals in the patch, total flower cover, and diversity of flowering plant species.

All models were computed in R v. 2.9.0 [29]. Maximal models were simplified in a manual stepwise backward selection on the basis of *F*-tests [30]. Predictors with *p* < 0.05 were considered to be significant. Predictors with *p* > 0.1 were removed from the maximal models. We transformed bee diversity and abundances recorded during transect walks to meet the assumptions of constant error variance and normality of errors (log_10_(*x* + 1)).

## 3. Results

### (a) Study 1: bees in semi-natural grasslands

We recorded 684 solitary bees (44 species), 49 bumble-bees (eight species) and 12 honeybees on 33 study grasslands.

**Figure 2.**
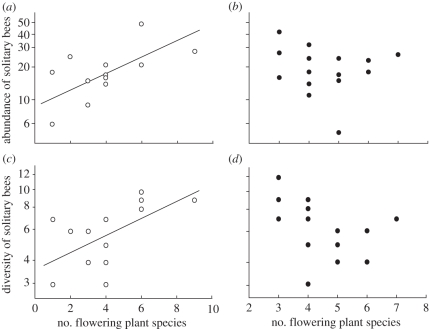
Effect of the diversity of flowering plants on (*a*,*b*) diversity and (*c*,*d*) abundance of solitary bees in grassland isolated from (*a*,*c*) oilseed rape (OSR) and (*b*,*d*) adjacent to OSR. Regression lines indicate significant relationships (*p* < 0.05). Model equations and *p*-values are given in the electronic supplementary material, appendix S3.

The number of flowering plant species in the grassland and the presence of adjacent OSR contributed to the explanation of diversity and abundance of solitary bees in grasslands. An interaction between the factors indicated that observed diversity and abundance of solitary bees increased with increasing number of flowering plants in grasslands isolated from OSR, but not in grasslands adjacent to OSR fields (table 1 and figure 2; equations in the electronic supplementary material, appendix S3).

**Table 1.** Landscape and local effects on bee diversity and abundances in grasslands and oilseed rape (OSR). Results are from ANCOVAs. Landscape-scale predictors were %OSR fields and %semi-natural habitats in 1 km radius. Local predictors were diversity of flowering plant species and presence of OSR (grasslands adjacent to OSR versus grasslands isolated from OSR) for bees in grasslands, and OSR field size and presence of grassland (OSR adjacent to grassland versus OSR isolated from grassland) for bees in OSR. Predictors are shown when *p* < 0.1 or when they are part of a significant interaction.

	d.f.	MS	*F*	*p*
study 1: bees in grasslands				
*abundance of solitary bees*				
flower diversity	1	0.19	5.1	0.032
presence of OSR	1	<0.01	<0.1	0.958
flower diversity × presence of OSR		0.13	3.5	0.072
residuals	28	0.04		
*abundance of bumble-bees*				
%OSR (1 km)	1	0.15	3.1	0.090
residuals	30	0.05		
*diversity of solitary bees*				
flower diversity	1	0.04	2.7	0.109
presence of OSR	1	0.01	0.8	0.382
%OSR (1 km)	1	0.04	2.9	0.098
flower diversity × presence of OSR	1	0.16	11.9	0.002
residuals	26	0.36		
study 2: bees in OSR fields				
*abundance of solitary bees*				
OSR field size	1	1.74	10.0	0.003
presence of grassland	1	0.67	5.7	0.023
residuals	31	0.11		
*abundance of bumble-bees*				
%OSR (1 km)	1	0.71	7.1	0.012
residuals	32	3.19		
*abundance of honeybees*				
none				
*diversity of bees*				
OSR field size	1	0.12	3.7	0.062
presence of grassland	1	0.19	6.0	0.020
%OSR (1 km)	1	0.30	9.8	0.004
residuals	30	0.03

The abundance of bumble-bees marginally decreased with increasing proportion of OSR in the landscape (*p* = 0.090; log (*y* + 1) = 0.042 + 0.011 × %OSR; table 1).

As the abundance of honeybees was very low, only presence–absence data were analysed. Honeybees were more likely to be found in grasslands adjacent to OSR (on 5 of 17 grasslands) than in isolated grasslands (0 of 16 grasslands; logistic regression, *χ*^2^ = 7.4, *p* = 0.006).

### (b) Study 2: bees in oilseed rape

We recorded 373 solitary bees belonging to 35 species, 92 bumble-bees belonging to nine species and 1080 honeybees in 34 OSR fields. Half of the species were only found in OSR adjacent to grassland but not in isolated OSR, and two species were found in isolated OSR but not in grasslands.

**Figure 3.**
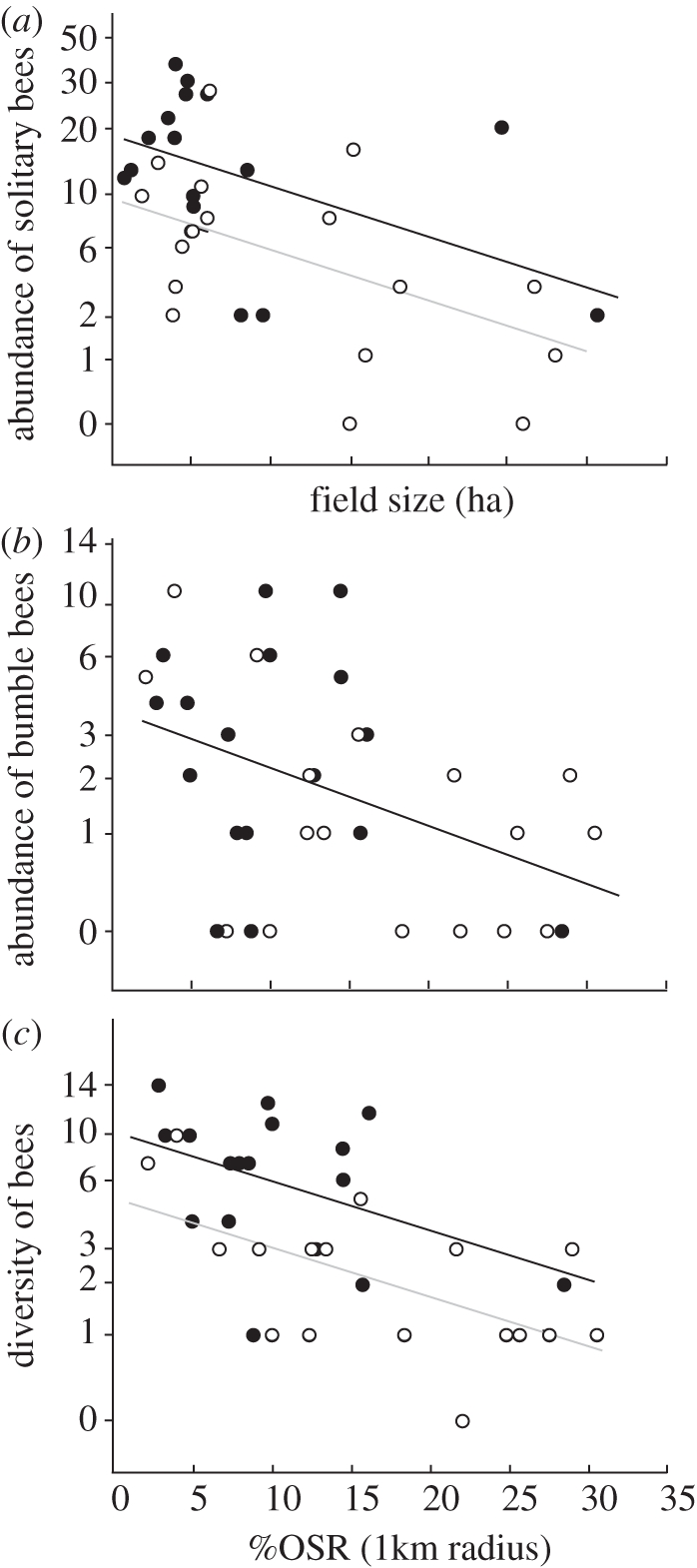
Effects of the presence of grassland (oilseed rape isolated from grassland versus oilseed rape adjacent to grassland), field size and percentage of oilseed rape (%OSR) within 1 km radius on bees in OSR. (*a*) Effect of field size (ha) on abundances of solitary bees; (*b*) effect of %OSR within 1 km radius on abundances of bumble-bees; (*c*) effect of %OSR within 1 km radius on bee diversity. Bee abundances are bee densities per 400 m^2^ and 60 min. Two regression lines are shown when bee abundance or diversity differed between OSR adjacent to grassland and OSR isolated from grassland. (*a*) log (*y* + 1) = 0.979 − 0.023 × field size, log (*y* + 1) = 1.268 − 0.023 × field size; (*b*) log (*y* + 1) = 0.679 − 0.017 × %OSR; (*c*) log (*y* + 1) = 0.923 − 0.016 × %OSR, log (*y* + 1) = 1.103 − 0.016 × %OSR. Black lines and black circles, OSR adjacent to grassland; grey lines and white circles, OSR isolated from grassland.

The presence of nearby grassland enhanced the abundance of solitary bees and the diversity of bees, but not the abundance of bumble-bees (table 1 and figure 3). A mean of 6.6 solitary bee individuals and 4.1 species of wild bees was found in isolated OSR, and 15.3 solitary bee individuals and 8.6 species of wild bees in OSR adjacent to grassland.

The abundance of solitary bees increased with the decreasing amount of OSR at the local scale (OSR field size; table 1 and figure 3*a*). The abundance of bumble-bees and bee diversity increased with decreasing %OSR at the landscape scale (table 1 and figure 3*b*,*c*). Honeybees were not influenced by any of the predictors.

### (c) Study 3: seed set of *P. veris*

**Figure 4.**
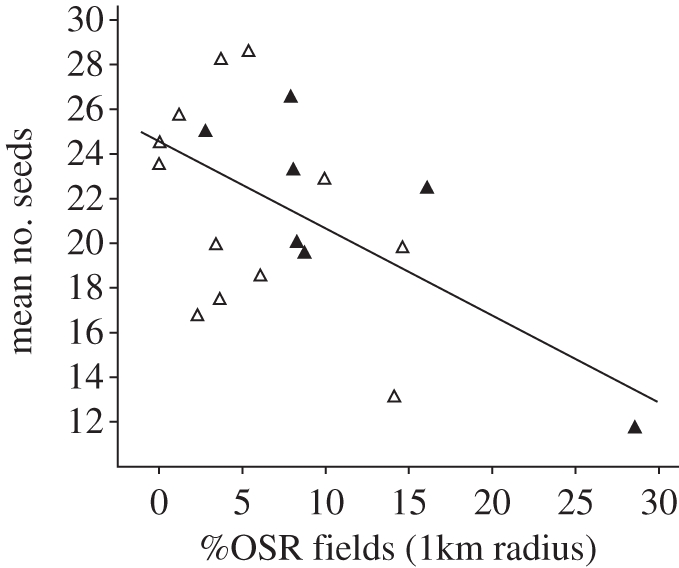
Relationship between percentage of oilseed rape (%OSR) in landscape sectors with 1 km radius and the reproductive success of *Primula veris* in grasslands, as mean number of seeds per fruit (simple regression: *n* = 19, *F* = 10.3, *p* = 0.005). Reproductive success did not differ between grasslands adjacent to oilseed rape and isolated grasslands (for results see text). Black triangles, grassland adjacent to OSR field; white triangles, grassland isolated from OSR field.

The number of seeds per fruit and seed weight per fruit of *P. veris* decreased with increasing %OSR in the surrounding landscape (figure 4; linear regression models: number of seeds, *F*_1,17_ = 10.3, *p* = 0.005; seed weight per fruit, *F*_1,17_ = 8.2, *p* = 0.011). The presence of adjacent OSR and local variables (number of *P. veris* individuals in the patch, total flower cover, flower diversity) did not have a significant effect on seed number or seed weight (all *p* > 0.1).

We recorded 31 bumble-bees in the 19 grasslands with *P. veris* during the transect walks of study 1 (mean ± s.e.: 1.38 ± 0.35 in isolated grasslands, 1.86 ± 0.46 in grasslands adjacent to OSR). Eight bee individuals (*Anthophora plumipes*, *Bombus* sp.) and two bombyliid flies were observed visiting *P. veris*. Abundances of bumble-bees and *P. veris* visitors were too low to detect significant relationships between pollinator abundance and seed number or seed weight.

## 4. Discussion

We conducted three studies to assess potential positive or negative effects of interactions between a mass-flowering crop and protected semi-natural grasslands on bees and native plants. Our results show that interactions between these habitats occur at different spatial scales, alter resource use of pollinators and reduce plant reproduction in conservation areas.

### (a) Study 1: bees in semi-natural grasslands

Diversity and abundance of solitary bees in grasslands isolated from OSR were enhanced by local flower diversity. In grasslands adjacent to OSR, diversity and abundance of solitary bees were independent of local flower diversity, and were high even in grasslands with low flower diversity. This suggests that bees even visited low-diversity grasslands if OSR was nearby providing rich floral resources.

Local abundance and diversity of flowering plants have often been found to be important drivers of bee abundance and diversity (e.g. [31,32]). However, there is some evidence that the relationship between local habitat factors and bees is affected by the amount of bee habitats in the surrounding [32,33]. Here we show that the importance of local resource availability for bees decreases as the availability of alternative flower resources at a larger spatial scale increases. We conclude that bee conservation measures, which aim to enhance local food availability in agricultural landscapes (e.g. restoration of high flower diversity in grasslands, organic farming or flower margin strips), are not only more efficient in crop-dominated landscapes than in landscapes dominated by non-crop habitats [32,33], but can be expected to be most needed and most efficient in crop landscapes where mass-flowering crops are not abundant.

In addition to local effects of adjacent OSR, we found weak landscape-scale effects of OSR, resulting in a decrease in bumble-bee abundances with increasing %OSR in the landscape. The number of bumble-bees recorded in grasslands was very low (49, compared with 684 solitary bees) and might explain why the OSR effect on bumble-bees was only marginally significant (*p* < 0.1). However, even moderate dilution of bumble-bees over the landscape during OSR flowering might have severe effects on bumble-bee-dependent plants (study 3), because abundances of bumble-bees are generally low at that time of the year [26].

### (b) Study 2: bees in oilseed rape

Bee diversity and the abundance of solitary bees, but not of bumble-bees and honeybees, were higher in OSR adjacent to grasslands than in isolated OSR. In contrast to solitary bees, bumble-bees and honeybees do not strongly depend on semi-natural habitats, because they are not as strongly constrained by their nesting requirements [22]. The contrasting habitat requirements resulted in altered proportions of solitary bees, bumble-bees and honeybees in OSR compared with grasslands. In our study, only 1.6 per cent of the bees in grasslands, but 68.6 per cent of the bees in OSR, were honeybees. Only 7.5 per cent of the wild bees (i.e. excluding honeybees) in grasslands, but 20 per cent of the wild bees in OSR fields, were bumble-bees. These shifts highlight the importance of OSR as attractive food resources, which can be exploited by pollinators that are not particularly restricted to semi-natural grasslands owing to low mobility and nesting requirements.

Bumble-bee abundances in OSR fields decreased with increasing %OSR in the landscape. Recent studies showed that generalist bumble-bees strongly benefit from OSR and other mass-flowering crops, although the benefit might differ between bumble-bee species [7]. Two to four months after colony establishment and after OSR flowering, colonies were larger and forager abundances were higher if the %OSR at a landscape scale was high [34–36]. In contrast to these studies showing a long-term numerical response of bumble-bee populations, our data (which were collected *during* OSR flowering) suggest a landscape-scale transient dilution of foraging bumble-bees. The dilution of bumble-bees can be considered to indicate that bumble-bee abundances declined at a landscape scale. It indicates that the distribution of bumble-bees in the landscape changed. The dilution of bumble-bees in the landscape might result in a decrease in pollination service per area unit of OSR, because OSR pollination and seed set depend at least partly on bee abundance [37,38]. In the long run, mass-flowering crops can enhance abundances of generalist pollinators and their pollination service at a landscape scale [35].

### (c) Study 3: seed set of *Primula veris*

The number of seeds per fruit of the grassland plant *P. veris* declined with increasing %OSR in the landscape. Our results indicate that OSR fields withdrew pollinators from *P. veris*. Competition for pollinators may occur when simultaneously flowering plant species are more attractive than the focal species, and the focal species suffers from pollen limitation [20]. Negative effects of co-flowering plant species on flower visitation and reproductive success of a focal species are known from neighbouring plants within a habitat [18–20], but have never been shown for co-flowering crops and native plants, nor for co-flowering plant species interacting over scales larger than local and across habitat borders. Introduced plant species have been hypothesized repeatedly to reduce flower visitation, pollen deposition and reproductive success of neighbouring native species [18,20].

OSR meets the criteria for being a strong competitor with *P. veris* for pollinators. (i) It is very attractive to pollinators because its floral density is huge. (ii) Bumble-bees, which are the main pollinators of *P. veris*, also forage in OSR, resulting in dilution of bumble-bees in OSR-dominated landscapes (study 2). (iii) Bumble-bee abundances are low at the time of *P. veris* and OSR flowering (study 1 [26]), which directly starts at the end of the hibernation period of bumble-bees, when queens are present but worker bumble-bees have not yet appeared. In addition to reduced visitation rates, OSR might reduce deposition of conspecific pollen on *P. veris* if pollinators visit flowers of both OSR and *P. veris* during the same foraging flight [17,39].

### (d) Implications

The current expansion of bee-attractive biofuel crops will increase cross-habitat exchanges of bees and competition between OSR and wild plants for pollinators. Spill-over effects of bees from semi-natural nesting habitats to crop habitats and bee-mediated spill-over of food resources from crop to nesting habitats may have a strong impact on population dynamics of bees and plants that depend on pollinators. Although there has been little additional evidence up to now, similar spill-over effects connecting crop and natural habitats can be expected for many types of species interactions in landscapes where highly productive sites and less productive, more natural sites co-occur.

Plants that depend on pollination by solitary bees might benefit from enhanced bee abundances at two temporal scales. In the year of OSR flowering, OSR might enhance the reproduction of plants in nearby grasslands with naturally low flower and bee diversity by attracting additional bees to those grasslands. In the year after OSR flowering, plant reproduction might be enhanced with a time lag owing to enhanced bee population abundances. From one year to the next, crop rotations can completely change the %OSR in a landscape and the crops cultivated in the vicinity of nature reserves. Thus, declines in plant reproduction in the presence of OSR in one year might be compensated for by an increased number of bee offspring in the following year.

Furthermore, given that bees live longer than the flowering period of OSR, plants in the vicinity of OSR may benefit from enhanced bee abundances *after* the period of OSR flowering. However, such beneficial effects might be restricted to plants pollinated by resource generalists, and could potentially increase competition between generalist and specialist pollinators in conservation areas.
